# Effects of angiotensin II receptor blockers on serum levels of epoxyeicosatrienoic acids and dihydroxyeicosatrienoic acids in patients admitted to a cardiovascular center

**DOI:** 10.1007/s00228-020-03061-1

**Published:** 2021-01-06

**Authors:** Yuka Kato, Asuna Senda, Yuji Mukai, Miki Yamashita, Yuki Sasaoka, Minayo Hanada, Fuminori Hongo, Mitsugu Hirokami, Anders Rane, Nobuo Inotsume, Takaki Toda

**Affiliations:** 1 Department of Clinical Pharmacology, Faculty of Pharmaceutical Sciences, Hokkaido University of Science, Sapporo, Japan; 2 Department of Clinical Pharmaceutics, Faculty of Pharmaceutical Sciences, Hokkaido University of Science, Sapporo, Japan; 3 Department of Pharmacy, Teine Keijinkai Hospital, Sapporo, Japan; 4 Department of Pharmacy, Sapporo Keijinkai Rehabilitation Hospital, Sapporo, Japan; 5 Cardiovascular Center, Teine Keijinkai Hospital, Sapporo, Japan; 6 Division of Clinical Pharmacology, Department of Laboratory Medicine, Karolinska University Hospital, Karolinska Institutet, Stockholm, Sweden; 7 Nihon Pharmaceutical University, Saitama, Japan

**Keywords:** Angiotensin II receptor blockers, Epoxyeicosatrienoic acids, Dihydroxyeicosatrienoic acids, Serum levels, Multiple linear regression analysis

## Abstract

**Purpose:**

Several clinical studies have demonstrated that angiotensin-converting enzyme inhibitors, but not angiotensin II receptor blockers (ARBs), reduce the risk of non-fatal myocardial infarction and cardiovascular mortality. We found that ARBs inhibited the activity of various cytochrome enzymes in arachidonic acid metabolism, resulting in decreased *in vitro* production of epoxyeicosatrienoic acids (EETs), which exhibit vasodilation and anti-inflammatory effects, and their subsequent metabolites, dihydroxyeicosatrienoic acids (DHETs). The present study examined the effects of ARBs on serum levels of EETs and DHETs in patients admitted to a cardiovascular center.

**Methods:**

A total of 223 patients were enrolled, of which 107 were exposed to ARBs in this study. ARB-free individuals were defined as the control group (*n* = 116). Serum levels of EETs and DHETs were measured by liquid chromatography–tandem mass spectrometry. Multiple linear regression analyses were carried out to identify covariates for total serum levels of EETs and DHETs.

**Results:**

A significant negative association was observed between ARB use and serum EET and DHET levels (*p* = 0.034), whereas a significant positive association was observed between the estimated glomerular filtration rate (eGFR) and serum EET and DHET levels (*p* = 0.007). The median serum total EET and DHET level in the ARB group tended to become lower than that in the control group, although the difference was not significant.

**Conclusion:**

ARB use and eGFR were significantly associated with total serum levels of EETs and DHETs. Our results suggest that ARBs could affect the concentration of EETs *in vivo*.

**Supplementary Information:**

The online version contains supplementary material available at 10.1007/s00228-020-03061-1.

## Introduction

Cytochromes P450 (CYPs), 2C8, 2C9, and 2J2 metabolize arachidonic acid (AA) to four regioisomeric epoxyeicosatrienoic acids (EETs), 14,15-, 11,12-, 8,9-, and 5,6-EET [1]. EETs act as autocrine and paracrine mediators [2] and exert vasodilatory [3] and anti-inflammatory activities [4]. EETs are subsequently metabolized by soluble epoxide hydrolase (sEH) [1] to the corresponding dihydroxyeicosatrienoic acids (DHETs), which are generally less biologically active compared to EETs [2], although Oltmann et al. [5] and Lu et al. [6] reported that DHETs exhibit more potent vasodilatory effects than EETs.

Several studies have reported that the activity of CYP2C8 and CYP2J2 and their polymorphic features constitute a group of enzymes with diminished enzyme activity that is involved in the development of cardiovascular diseases [7–10]. Oni-Orisan et al. [11] recently showed that patients with obstructive coronary artery disease (CAD) have lower levels of plasma EETs and total EETs and DHETs than patients without apparent CAD. Low levels of circulating total EETs and DHETs may theoretically increase the risk of cardiovascular events.

Angiotensin II receptor blockers (ARBs) are the recommended initial treatment for hypertension according to the 2017 ACC/AHA [12] and 2018 ESC/ESH [13] guidelines. Several clinical trials have suggested that ARBs and angiotensin-converting enzyme inhibitors (ACEIs) have a prophylactic effect against cardiovascular events [14–20]. However, several recent large-scale meta-analyses reported that ARBs have limited efficacy in preventing cardiovascular events [21–23]. Hoang et al. [21] reported that ACEIs (but not ARBs) reduce the risks of non-fatal myocardial infarction (MI) and cardiovascular mortality in CAD patients. Similar results have been reported in patients with heart failure [22] and diabetes mellitus [23].

We previously demonstrated that ARBs inhibit AA metabolism via CYP enzymes *in vitro*, with differing degrees of inhibition among ARBs [24, 25]. Telmisartan exhibited the most potent inhibitory effect on AA metabolism among the seven ARBs investigated, whereas candesartan exhibited little inhibitory effect [25]. Similar results have been reported by other researchers [26, 27]; however, it remains unclear whether ARBs reduce the production of EETs from AA *in vivo*.

Given this background, we investigated the effects of ARBs on serum levels of EETs and DHETs in patients admitted to a cardiovascular center. We also explored the covariates related to total EET and DHET serum levels using multiple linear regression analysis.

## Materials and methods

### Study design

A total of 223 patients admitted to the Cardiovascular Center at Teine Keijinkai Hospital between October 2013 and October 2017 were enrolled in the study. Individuals continuously exposed to an ARB (azilsartan, candesartan, losartan, irbesartan, olmesartan, telmisartan, or valsartan) for at least 4 weeks were assigned to the ARB group (*n* = 107). Individuals who had not taken any ARB were assigned to the control group (*n* = 116). Informed consent was obtained from each participant included in the study. The study protocol was approved by the Ethics Committee of Teine Keijinkai Hospital. All procedures of this study were in accordance with the ethical standards of the institutional research committee (Ethics Committee of Teine Keijinkai Hospital, 2013-043) and the 1964 Helsinki declaration and its later amendments or comparable ethical standards.

Serum concentrations of EETs and DHETs were determined using residual serum collected for biochemical examinations. Serum samples containing 0.2 mg/mL of butylated hydroxytoluene in 50% methanol (final concentration: 3.9 μg/mL) as an antioxidant were frozen at − 30 °C at Teine Keijinkai Hospital and then transported to Hokkaido University of Science, where they were stored at − 80 °C until analysis. Concentrations of EETs and DHETs in serum were measured within 1 week of collection.

### Chemicals

Eight eicosanoids (14,15-, 11,12-, 8,9-, and 5,6-EET, and 14,15-, 11,12-, 8,9-, and 5,6-DHET) and their corresponding deuterated eicosanoids (14,15-, 11,12-, 8,9-, and 5,6-EET-d_11_, and 14,15-, 11,12-, and 8,9-DHET-d_11_) as internal standards were purchased from Cayman Chemical (Ann Arbor, MI), except for 5,6-DHET-d_11_, which was commercially unavailable. Therefore, 8,9-DHET-d_11_ was used as an internal standard for the determination of 5,6-DHET. OASIS® HLB solid-phase extraction cartridges (3 cc) were purchased from Waters (Milford, MA). All other chemicals and solvents were HPLC or special grade.

### Sample preparation

An aliquot of 250 μL of serum was mixed with 50 μL of internal standard solution and 950 μL of ethanol and placed on ice for 20 min. The mixture was then centrifuged at 6490×*g* for 5 min. The resulting supernatant was loaded onto a preconditioned OASIS® HLB cartridge, and EETs and DHETs were extracted using ethyl acetate. The eluate was evaporated to dryness and reconstituted with 55 μL of 50% acetonitrile. After centrifugation at 6490×*g* for 5 min, an aliquot of 40 μL of the supernatant was used for liquid chromatography–tandem mass spectrometry (LC-MS/MS). Samples were analyzed in duplicate for each subject.

### LC-MS/MS conditions

Serum concentrations of EETs and DHETs were determined using an LC-MS/MS method described previously [24, 28]. The LC-MS/MS system consisted of an Agilent 1200 series HPLC (Agilent Technologies, Santa Clara, CA) coupled to a QTRAP® API3200 mass spectrometer (AB Sciex, Framingham, MA). Separation of EETs and DHETs was conducted at 50 °C using an Ascentis Express C18 column (2.7-μm particle size, 10 cm × 2.1 mm; Sigma-Aldrich, St. Louis, MO). Mobile phases A and B consisted of 0.1% formic acid in acetonitrile and water, respectively. The flow rate was set at 0.3 mL/min. The gradient program was as follows: 50% B for 27 min, 50–90% B from 27 to 28 min, 90% B from 28 to 35 min, 90–50% B from 35 to 36 min, and re-equilibration at 50% B from 36 to 43 min. Electrospray ionization was employed to determine EETs and DHETs by multiple reaction monitoring in negative ion mode. Lower limit of quantification (LLOQ) values for each EET and DHET concentration with a signal/noise ratio > 10 were as follows; 0.35 nM (0.11 ng/mL), 0.11 (0.036), 3.6 (1.2), 0.20 (0.064), 0.13 (0.043), 0.13 (0.043), 0.10 (0.034), and 0.077 (0.026) for 14,15-, 11,12-, 8,9-, and 5,6-EET and 14,15-, 11,12-, 8,9-, and 5,6-DHET, respectively.

### Data analysis

Patient characteristics were compared using chi-squared or unpaired *t* tests, whereas the Mann-Whitney test was used to analyze differences in levels of EET and DHET regioisomers as well as total levels of EETs and DHETs between the ARB and control groups. Multiple linear regression analyses adjusting for age, sex, body mass index (BMI), smoking status, estimated glomerular filtration rate (eGFR), history of MI, and medications (including ARBs, calcium channel blockers [CCBs], ß-blockers, diuretics, HMG-CoA reductase inhibitors [statins], antiplatelets, anticoagulants, and antidiabetics) were performed using SPSS software (ver. 24.0, IBM Japan, Tokyo, Japan). Patient characteristics and serum levels of EETs and DHETs are expressed as the mean ± SD and median, respectively.

## Results

### Patient characteristics

Patient characteristics are shown in Table 1. There were no significant differences between groups with respect to age, sex, systolic blood pressure, smoking status, eGFR, alanine aminotransferase, or history of MI. BMI was significantly lower in the control group than in the ARB group. Diastolic blood pressure (DBP) and aspartate aminotransferase (AST) were significantly higher in the control group compared to the ARB group. CCBs, diuretics, and antiplatelets were prescribed less frequently to the control group.

**Table 1:** Patient characteristics

	Control (*n* = 116)	ARB (*n* = 107)	*p* value
Age (years)	69.4 ± 11.2	72.1 ± 9.8	0.067^a^
Sex (male/female)	81/35	63/44	0.088^b^
BMI (kg/m^2^)	23.4 ± 4.0	25.2 ± 3.8	0.001^a^
SBP (mmHg)	124.4 ± 18.4	127.3 ± 17.8	0.244^a^
DBP (mmHg)	72.5 ± 11.4	67.4 ± 13.3	0.002^a^
Smoking status (current-/ex-/non-smoker)	20/31/55	13/32/46	–
eGFR (mL/min)	64.2 ± 23.8	59.4 ± 24.3	0.135^a^
AST (IU/L)	28.4 ± 11.2	25.5 ± 9.0	0.036^a^
ALT (IU/L)	24.0 ± 13.3	21.9 ± 12.6	0.227^a^
History of MI	17	23	0.183^b^
Medication (%)			
CCBs	36 (31)	71 (66)	< 0.001^b^
β-Blockers	50 (43)	47 (44)	0.902^b^
Diuretics	18 (16)	36 (34)	0.002^b^
Statins	58 (50)	66 (62)	0.079^b^
Antiplatelets	66 (57)	75 (70)	0.041^b^
Anticoagulants	31 (27)	31 (29)	0.708^b^
Antidiabetics	23 (20)	30 (28)	0.150^b^

Mean ± SD, ^a^unpaired t-test, ^b^chi-squared test

*ARBs* angiotensin II receptor blockers, *BMI* body mass index, *SBP* systolic blood pressure, *DBP* diastolic blood pressure, *eGFR* estimated glomerular filtration rate, *AST* aspartate aminotransferase, *ALT* alanine aminotransferase, *MI* myocardial infarction, *CCBs* calcium channel blockers; Statins: HMG-CoA reductase inhibitors

### EET and DHET serum levels

Table 2 summarizes EET and DHET serum levels for each group. A large number of the EET serum levels were below the LLOQ; in particular, 8,9-EET could not be detected in any sample in either group. Serum levels of 14,15-EET and 8,9-DHET in the ARB group were significantly lower than those in the control group. The EET/DHET ratio, an indicator of sEH activity, exhibited large interpatient variability.

**Table 2:** EET and DHET serum levels in the control and ARB groups

Control (*n*=116)	EET (nM) (number of samples above LLOQ)	DHET (nM)(number of samples above LLOQ)	EET/DHET (number of calculable samples)
14,15-form	2.60 [0.88–5.21] (8)	0.47 [0.15–1.25] (116)	3.43 [1.07–14.3] (8)
11,12-form	0.84 [0.12–3.05] (28)	0.38 (0.13–1.13] (116)	2.20 [0.22–7.83] (28)
8,9-form	ND (0)	0.25 [0.13–0.74] (61)	– (0)
5,6-form	1.59 [0.20–12.1] (33)	0.83 [0.08–7.86] (63)	1.44 [0.68–12.9] (20)
Total	2.46 [0.20–16.4] (36)	1.40 [0.31–9.43] (116)	0.95 [0.16–21.5] (36)
ARB (*n*=107)			
14,15-form	0.81^†^ [0.35–2.20] (6)	0.46 [0.13–1.00] (107)	1.35 [0.38–4.31] (6)
11,12-form	0.68 [0.11–1.39] (17)	0.41 [0.13–1.01] (105)	1.57 [0.21–2.78] (17)
8,9-form	ND (0)	0.23* [0.10–0.58) (49)	– (0)
5,6-form	1.21 [0.30–4.32] (20)	0.65 [0.08–5.61] (54)	2.00 [0.08–8.49] (9)
Total	1.91 [0.32–7.74] (22)	1.23 [0.27–6.84] (107)	1.14 [0.08–7.30] (22)

Median [range]

Control (*n* = 116)

Median [range]

ARB (*n* = 107)

^†^*p* < 0.01, *p < 0.05 vs control group, Mann-Whitney *U* test

*EETs* epoxyeicosatrienoic acids, *DHETs* dihydroxyeicosatrienoic acids, *LLOQ* lower limit of quantification, *ND* not detected

### Multiple linear regression analysis

Prior to multiple linear regression analysis, single linear regression analysis was applied to broadly estimate covariates and confirmed that ARB and antiplatelets use, age, and eGFR significantly affected total EET and DHET serum levels (Supplementary Table S1). ARB use and eGFR were considered as covariates for calculating total EET and DHET serum levels in the multiple linear regression analysis (Table 3). A significant negative association was observed between ARB use and total EET and DHET serum levels (*p* = 0.034), whereas a significant positive association was observed between eGFR and serum levels (*p* = 0.007).

**Table 3:** Results of the multiple linear regression analysis exploring the covariates related to total EET and DHET serum levels

Variable	Coefficient	SE	*p* value
Total EETs + DHETs			
Intercept	1.470	0.618	0.018
eGFR	0.023	0.009	0.007
ARB use	− 0.878	0.411	0.034

Adjusted *R*^2^: 0.055

*SE* standard error, *EETs* epoxyeicosatrienoic acids, *DHETs* dihydroxyeicosatrienoic acids, *eGFR* estimated glomerular filtration rate, *ARB* angiotensin II receptor blocker

### Total EET and DHET serum levels

**Fig. 1:**
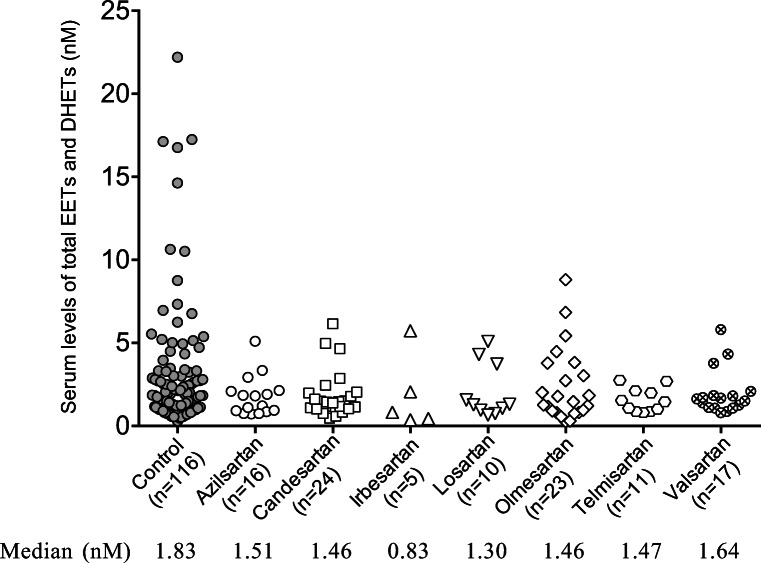
Serum levels and median total EET and DHET levels in the control and ARB groups. *EETs* epoxyeicosatrienoic acids, *DHETs* dihydroxyeicosatrienoic acids

Figure 1 summarizes the median values for total EET and DHET serum levels for each group. The median total EET and DHET serum level in the ARB group (1.47 nM) tended to become lower than that in the control group (1.83 nM), although the difference was not significant. The adjusted *R*^*2*^ (coefficient of determination) value for the multiple regression analysis indicated that eGFR and ARB use contributed only 5.5% to the change in total EET and DHET serum levels (Table 3).

## Discussion

Serum levels of DHETs, the active metabolites of EETs, decline with decreasing EET levels and affect homeostasis of the cardiovascular system [2, 5, 6]. Clinical studies should therefore assess the effects of medications on serum EET and DHET concentrations. A large number of EET serum levels were below the LLOQ (Table 2), whereas total EET serum levels were higher than total DHET levels. These results were consistent with a previous report [29]. The 14,15-EET concentrations were significantly reduced in patients taking ARBs, but over 90% of samples were below the LLOQ. 8,9-DHTE levels were significantly reduced in patients taking ARBs, but the difference in the median values was small. There were no significant differences in the levels of other eicosanoids or the EET/DHET ratio in patients taking ARBs. The EET/DHET ratio, which indicates sEH activity, was calculated to be 0.310 (36/116) and 0.206 (22/107) in the control and ARB groups, respectively, with large interpatient variability. As the sum of EET and DHET levels is thought to represent EET biosynthesis, the effect of ARBs on reducing EET production from AA via the CYP pathway could be described by the total EET and DHET serum levels.

The other clinical conditions of patients who participated in this study varied, with the exception of taking ARBs. This study examined changes in serum eicosanoid levels due to ARB use with ethical considerations but did not conduct any interventions in routine medications. It would be useful to investigate the effects of ARBs on the biokinetics of eicosanoids in clinical trials that further classify patient groups.

As shown in Table 1, BMI was significantly lower in the control group than in the ARB group, whereas DBP and AST were significantly higher in the control group than in the ARB group. The differences in BMI and DBP between the two groups were < 10%, and the mean AST level in both groups was within the normal range (7–38 IU/L). Therefore, these differences, although significant, would have little influence on the results of this study. CCBs, diuretics, and antiplatelets were co-prescribed more frequently to patients in the ARB group. All patients enrolled in this study were admitted to Cardiovascular Center at Teine Keijinkai Hospital, but the patients in the control group were not always treated for hypertension. The glucuronate conjugate of clopidogrel, a commonly prescribed antiplatelets, exhibits a time-dependent inhibitory effect on CYP2C8 [30, 31]. Clopidogrel was prescribed to 32.8% (38/116) of patients in the control group and 40.1% (43/107) of patients in the ARB group (*p* = 0.249, chi-squared test). Other than clopidogrel, there was no concomitant use of CCBs, diuretics, and antiplatelets that could affect the metabolism of EETs. Therefore, concomitant drug use would also have a negligible effect on the results of this study.

ARB use was the covariate for calculating the serum levels of total EETs and DHETs (Table 3). The median total EET and DHET serum level in the ARB group tended to become lower than that in the control group, although the difference was not significant (Fig. 1). These results are consistent with studies demonstrating an inhibitory effect of ARBs on AA metabolism *in vitro* [24, 25].

Renal function was also associated with total EET and DHET serum levels (Table 3). A significant negative correlation was observed between eGFR and age (Spearman’s rank correlation coefficient *ρ* = − 0.552, *p* < 0.001). Kawabata et al. [32] reported decreased blood concentrations of AA metabolites in geriatric patients. Although the concentrations of EETs and DHETs in urine were not assessed in this study, the positive relationship between eGFR and serum levels of EETs and DHETs could reflect an age-associated decrease in production rather than delayed excretion due to decreased renal function.

The enzymatic activities of CYPs and sEHs are regulated by female hormones/estrogens, resulting in a gender disparity in terms of EET-mediated effects [33]. Experimental human models and pathophysiological studies suggest that enzymes involved in EET biosynthesis and metabolism play a role in controlling blood pressure in a gender-specific manner. Female hormones would protect women from cardiovascular events until menopause, but the risk increases rapidly after menopause [34]. Multiple linear regression analyses in this study were conducted to calculate total EET and DHET serum levels regardless of gender. Seventy-four of 79 female participants in this study were aged over 60 years and considered menopausal, and they were thought to have as low female hormone levels as low as males, which is why gender was not included in the final model.

**Fig. 2:**
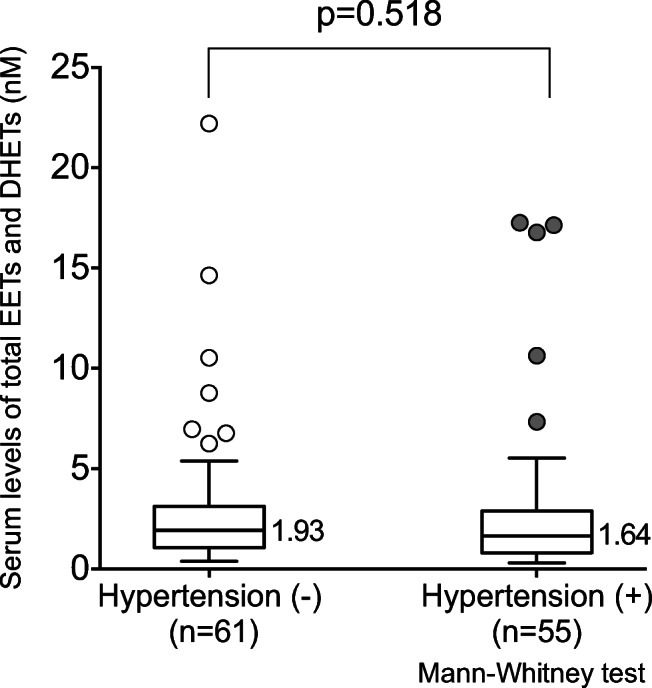
Serum levels of total EETs and DHETs in the control group and presence or absence of a diagnosis of hypertension. Data are presented as the median (midline), interquartile range (box), and 95% confidence intervals (whiskers). *EETs* epoxyeicosatrienoic acids, *DHETs* dihydroxyeicosatrienoic acids

Minuz et al. [29] reported lower levels of plasma EETs and total EETs and DHETs in patients with renovascular disease compared with essential hypertensive patients or healthy normotensive subjects. We did not find any significant difference regarding patients with hypertension (*p* = 0.518, Fig. 2), indicating that hypertension has a negligible effect on serum levels of total EETs and DHETs in this population.

Oni-Orisan et al. [11] reported a median plasma level of total EETs and DHETs in patients with obstructive CAD of 2.59 ng/mL, whereas the median serum levels of total EETs and DHETs in the ARB and control groups in this study were 0.497 and 0.620 ng/mL, respectively. The mean (± SD) age of patients in the ARB group (72.1 ± 9.8 years) in our study was higher than that reported by Oni-Oresan et al. (63.6 ± 10.2) [11]. As the AA concentration in blood decreases with age in humans [32], the discrepancy in circulating levels of EETs and DHETs between the two studies could be explained in part by the difference in mean age of the study populations.

The adjusted *R*^*2*^ value from the multiple regression analysis indicated that eGFR and ARB use contributed only 5.5% to the difference in the serum levels of total EETs and DHETs. These results suggest that as yet unknown additional factors affect the serum level of total EETs and DHETs.

In contrast to ARBs, ACEIs reportedly increase EET levels by inhibiting ACE and increasing bradykinin, which causes vascular relaxation via the release of endothelial relaxing factors, including EETs [35]. This may be related to observations from clinical trials suggesting that ACEIs have a prophylactic effect on cardiovascular events [14–17].

In conclusion, multiple linear regression analysis revealed that ARB use and eGFR are significantly associated with serum levels of EETs and DHETs. The median total EET and DHET serum level in the ARB group tended to become lower than that in the control group, although the difference was not statistically significant. Further studies are thus necessary to elucidate the relationship between serum levels of EETs and DHETs and the risk of cardiovascular events in patients taking ARBs.

## Supplementary Information

ESM 1 (DOCX 18 kb)

## Data Availability

Not applicable
