# Correction to: Effects of angiotensin II receptor blockers on serum levels of epoxyeicosatrienoic acids and dihydroxyeicosatrienoic acids in patients admitted to a cardiovascular center

**DOI:** 10.1007/s00228-021-03092-2

**Published:** 2021-01-23

**Authors:** Yuka Kato, Asuna Senda, Yuji Mukai, Miki Yamashita, Yuki Sasaoka, Minayo Hanada, Fuminori Hongo, Mitsugu Hirokami, Anders Rane, Nobuo Inotsume, Takaki Toda

**Affiliations:** 1 Department of Clinical Pharmacology, Faculty of Pharmaceutical Sciences, Hokkaido University of Science, Sapporo, Japan; 2 Department of Clinical Pharmaceutics, Faculty of Pharmaceutical Sciences, Hokkaido University of Science, Sapporo, Japan; 3 Department of Pharmacy, Teine Keijinkai Hospital, Sapporo, Japan; 4 Department of Pharmacy, Sapporo Keijinkai Rehabilitation Hospital, Sapporo, Japan; 5 Cardiovascular Center, Teine Keijinkai Hospital, Sapporo, Japan; 6 Division of Clinical Pharmacology, Department of Laboratory Medicine, Karolinska University Hospital, Karolinska Institutet, Stockholm, Sweden; 7 Nihon Pharmaceutical University, Saitama, Japan

**Correction to: European Journal of Clinical Pharmacology**

10.1007/s00228-020-03061-1

The article Effects of angiotensin II receptor blockers on serum levels of epoxyeicosatrienoic acids and dihydroxyeicosatrienoic acids in patients admitted to a cardiovascular center, written by Yuka Kato, Asuna Senda, Yuji Mukai, Miki Yamashita, Yuki Sasaoka, Minayo Hanada, Fuminori Hongo, Mitsugu Hirokami, Anders Rane, Nobuo Inotsume and Takaki Toda, was originally published electronically on the publisher’s internet portal on 6 January 2021 without open access. With the author(s)’ decision to opt for Open Choice the copyright of the article changed on 12 January 2021 to © The Author(s) 2021 and the article is forthwith distributed under a Creative Commons Attribution 4.0 International License, which permits use, sharing, adaptation, distribution and reproduction in any medium or format, as long as you give appropriate credit to the original author(s) and the source, provide a link to the Creative Commons licence, and indicate if changes were made. The images or other third party material in this article are included in the article’s Creative Commons licence, unless indicated otherwise in a credit line to the material. If material is not included in the article’s Creative Commons licence and your intended use is not permitted by statutory regulation or exceeds the permitted use, you will need to obtain permission directly from the copyright holder. To view a copy of this licence, visit http://creativecommons.org/licenses/by/4.0.

The Original article has been corrected.

