# Supplementary material for: Effects of angiotensin II receptor blockers on serum levels of epoxyeicosatrienoic acids and dihydroxyeicosatrienoic acids in patients admitted to a cardiovascular center
Source: Eur J Clin Pharmacol. 2021 Jan 6;77(6):887–94. doi: 10.1007/s00228-020-03061-1 (PMC8128744; doi:10.1007/s00228-020-03061-1)
Supplement: Supplementary file 1 — (DOCX 18 kb) [file 228_2020_3061_MOESM1_ESM.docx]

Article title: Effects of angiotensin II receptor blockers on serum levels of epoxyeicosatrienoic acids and dihydroxyeicosatrienoic acids in patients admitted to a cardiovascular center

Journal name: European Journal of Clinical Pharmacology

Author names: Yuka Kato^1^, Asuna Senda^1^, Yuji Mukai^1^, Miki Yamashita^2^, Yuki Sasaoka^3, 4^, Minayo Hanada^3^, Fuminori Hongo^3^, Mitsugu Hirokami^5^, Anders Rane^6^, Nobuo Inotsume^1, 7^, Takaki Toda^1^

Affiliation:

^1^ Department of Clinical Pharmacology, Faculty of Pharmaceutical Sciences, Hokkaido University of Science, Sapporo, Japan

^2^ Department of Clinical Pharmaceutics, Faculty of Pharmaceutical Sciences, Hokkaido University of Science, Sapporo, Japan

^3^ Department of Pharmacy, Teine Keijinkai Hospital, Sapporo, Japan

^4^ Department of Pharmacy, Sapporo Keijinkai Rehabilitation Hospital, Sapporo, Japan

^5^ Cardiovascular Center, Teine Keijinkai Hospital, Sapporo, Japan

^6^ Division of Clinical Pharmacology, Department of Laboratory Medicine, Karolinska University Hospital, Karolinska Institutet, Stockholm, Sweden

^7^ Nihon Pharmaceutical University, Saitama, Japan

e-mail address of the corresponding author: toda@hus.ac.jp (Takaki Toda)

Supplementary Table S1 Results of the single linear regression analysis exploring the covariates related to total EET and DHET serum levels

ARBs: Angiotensin II receptor blockers, BMI: body mass index, eGFR: estimated glomerular filtration rate, MI: myocardial infarction, CCBs: calcium channel blockers, Statins: HMG-CoA reductase inhibitors

| Variable | Coefficient | SE | p value |
| --- | --- | --- | --- |
| ARBs | −0.992 | 0.389 | 0.011 |
| Age | −0.050 | 0.018 | 0.006 |
| Sex | −0.153 | 0.412 | 0.710 |
| BMI | −0.023 | 0.049 | 0.635 |
| Smoking | 0.553 | 0.558 | 0.323 |
| eGFR | 0.025 | 0.008 | 0.002 |
| History of MI | −0.879 | 0.510 | 0.086 |
| Medication |  |  |  |
| CCBs | −0.366 | 0.394 | 0.353 |
| β-blockers | 0.376 | 0.397 | 0.344 |
| Diuretics | −0.368 | 0.459 | 0.424 |
| Statins | −0.091 | 0.396 | 0.820 |
| Antiplatelets | −0.852 | 0.405 | 0.036 |
| Anticoagulants | 0.060 | 0.440 | 0.891 |
| Antidiabetics | −0.245 | 0.463 | 0.596 |
